# Supplementary material for: Stakeholders’ Perceptions Regarding Adaptation and Implementation of Existing Individual and Environmental Workplace Health Promotion Interventions in Blue-Collar Work Settings
Source: Int J Environ Res Public Health. 2022 Oct 19;19(20):13545. doi: 10.3390/ijerph192013545 (PMC9603088; doi:10.3390/ijerph192013545)
Supplement: Supplementary file 1 [file ijerph-19-13545-s001.zip › ijerph-1934915-supplementary.pdf]

International Journal of
Environmental Research and Public Health

MDPI

Supplementary Materials. Key sample questions stakeholder interviews.

Topic	Sample Question
**(1) Advantages and disadvantages**	Can you share the advantages and disadvantages of implementing these interventions for you?
**(2) Implementation strategy**	Which tools do you need to implement these interventions?
**(3) Expectations and attitude towards the intervention**	Can you share why you would or would not want to implement these interventions?
**(4) Employee participation and support**	How can support for these interventions be created in your organisation?
**(5) Required resources**	Which resources do you need to implement these interventions?
**(6) Legislation and regulations**	To what extent do the interventions fit into your organisation's (human resources) policies and regulations?
